# Mutations of Key Functional Residues in CRM1/XPO1 Differently Alter Its Intranuclear Localization and the Nuclear Export of Endogenous Cargos

**DOI:** 10.3390/biom14121578

**Published:** 2024-12-10

**Authors:** Miren Josu Omaetxebarria, Maria Sendino, Liher Arrizabalaga, Irune Mota, Ana Maria Zubiaga, José Antonio Rodríguez

**Affiliations:** 1 Department of Biochemistry and Molecular Biology, University of the Basque Country (UPV/EHU), 48940 Leioa, Spain; mirenjosu.omaetxebarria@ehu.eus; 2 Department of Genetics, Physical Anthropology and Animal Physiology, University of the Basque Country (UPV/EHU), 48940 Leioa, Spain; maria.sendino@ehu.eus (M.S.); larrizabalaga004@ikasle.ehu.eus (L.A.); irune97@gmail.com (I.M.); ana.zubiaga@ehu.eus (A.M.Z.)

**Keywords:** CRM1, XPO1, Cajal body, nucleolus, nuclear export, mutagenesis

## Abstract

CRM1 (XPO1) has been well-characterized as a shuttling receptor that mediates the export of protein and RNA cargos to the cytoplasm, and previous analyses have pinpointed several key residues (A541, F572, K568, S1055, and Q742) that modulate CRM1 export activity. CRM1 also has a less studied nuclear function in RNA biogenesis, which is reflected by its localization to the Cajal body and the nucleolus. Here, we have investigated how the mutation of these key residues affects the intranuclear localization of CRM1 and its ability to mediate export of endogenous cargos. We identify A541K as a separation-of-function mutant that reveals the independent nature of the Cajal body and nucleolar localizations of CRM1. We also show that the F572A mutation may have strikingly opposite effects on the export of specific cargos. Importantly, and in contrast to previous claims, our findings indicate that S1055 phosphorylation is not generally required for CRM1 function and that the Q742 is not a function-defining residue in human CRM1. Collectively, our findings provide new insights into an understudied aspect of CRM1 biology and highlight several important issues related to CRM1 function and regulation that need to be re-evaluated and addressed in more detail.

## 1. Introduction

The nuclear export receptor CRM1/XPO1 (hereafter referred to as CRM1) regulates the nucleocytoplasmic distribution of dozens of human proteins and several classes of RNA molecules, reviewed in [1]. Nuclear export mediated by CRM1 influences multiple aspects of cellular physiology and is often altered in pathological states, such as cancer [2]. The overexpression of CRM1 is common in many types of solid tumors and hotspot CRM1 mutations, most frequently E571K, are detected in up to 25% of patients with specific types of hematologic malignancies [1]. The nuclear export activity of CRM1 can be pharmacologically inhibited by drugs, such as leptomycin B (LMB) [3], and is increasingly regarded as a relevant therapeutic target in cancer [2]. While the elevated in vivo toxicity of LMB precluded its clinical use, other less toxic CRM1 inhibitors have been developed, and one of these drugs, selinexor (KPT-330), has already been approved for the treatment of patients with multiple myeloma [4] and diffuse large B-cell lymphoma [5].

CRM1-mediated protein export is a widely studied and well-characterized process. In the nucleus, CRM1 recognizes cargo proteins that bear a so-called nuclear export signal (NES), a short amino acid motif with a characteristic pattern that includes four to six hydrophobic residues [6]. CRM1 binds NES motifs weakly, but the interaction is stabilized by nuclear RanGTP, leading to the formation of trimeric CRM1/cargo/RanGTP export complexes that exit the nucleus through the nuclear pore. Upon reaching the cytoplasmic side of the pore, GTP hydrolysis by Ran leads to dissociation of the export complex and to the release of the cargo in the cytoplasm [6]. The mechanistic details of CRM1-mediated nuclear export were elucidated in a series of structural studies showing that NES peptides dock into a cleft in the surface of the receptor (the so-called NES-binding groove) [7,8,9,10]. Importantly, the open/closed conformation of the groove (and thus, the binding and release of NES peptides) was found to be allosterically regulated by other CRM1 regions, most prominently its c-terminal end [11,12], which acts as an auto-inhibitory domain.

The detailed structural characterization of CRM1 nuclear export function, reviewed in [13], has been supported and extended by site-directed mutagenesis studies that have pinpointed key functional residues. Structure-guided mutagenesis confirmed that NES binding involves multiple hydrophobic interactions and salt bridges with several key amino acids in the NES-binding groove of CRM1. Mutations in these residues, such as A541K and F572A, severely impaired NES binding and export [9,10,14,15]. Another groove residue, K568, was subsequently reported to act as a “filter” that prevents binding of non-functional NES-like motifs to the receptor [16]. A mutation of this residue (K568A) was shown to abrogate this filter [16] but also to reduce the nuclear export of bona fide NES motifs [15]. More recently, an amino acid outside of the NES-binding groove, Q742, was described as an important function-defining residue for human CRM1 [17]. The mutation of this non-conserved residue to its yeast counterpart (Q742T) was reported to interfere with the nuclear export function of the human receptor [17].

Mutagenesis studies have also contributed to elucidate the mechanism of action of CRM1 inhibitors, such as LMB and selinexor. These compounds sterically block NES access to the NES-binding groove by covalently binding to CRM1 residue C528 [18]. Accordingly, the mutation C528S renders CRM1 insensitive to these drugs.

Finally, mutagenesis analyses have also provided information on how CRM1 nuclear export function can be regulated by post-translational modifications (PTMs), specifically by phosphorylation of S1055 in the c-terminal autoinhibitory domain [19,20]. A non-phosphorylatable mutant of CRM1 (S1055A) was reportedly unable to export several autophagy-related cargos to the cytoplasm [20]. Phosphorylation of this residue by the STK38 kinase has been claimed to be an essential requirement for CRM1 nuclear export activity [21]. Also, in the context of PTM-mediated regulation, the K568 residue has been consistently reported as a CRM1 acetylation site in multiple proteomics studies, as indicated in the PhosphositePlus repository [22]. However, it still unknown whether this potential PTM contributes to modulate CRM1 activity.

Importantly, there are still several outstanding questions on how CRM1-mediated nuclear export is affected by the mutants described above. For example, the effect of different NES binding groove mutations on the ability of CRM1 to export endogenous cargos remains to be further characterized. In addition, although the S1055A mutation has been reported to abrogate export of some cargos [20], it is still unclear to what extent phosphorylation of this residue is generally required for CRM1 nuclear export activity.

In addition to its canonical function as a nuclear export receptor, CRM1 also plays other, less well-characterized roles in crucial aspects of cell physiology, such as the biogenesis of important short non-coding RNA species inside the nucleus. In this regard, CRM1 regulates intranuclear steps during the metabolism of certain small nuclear RNAs (snRNAs) and small nucleolar RNAs (snoRNAs), which are crucial factors in splicing and ribosome biogenesis, respectively [23,24,25]. The maturation and assembly of these RNA molecules into functional ribonucleoproteins (snRNPs and snoRNPs) are complex, multistep processes that involve two specific compartments within the nucleus: the Cajal body (CB) and the nucleolus, reviewed in [26,27,28]. In HeLa cells, the participation of CRM1 in these processes is reflected by its dynamic localization to these two intranuclear compartments. Thus, CRM1 is present in CBs in untreated cells [24] and drastically relocates to the nucleolus when the activity of RNA polymerase I is inhibited by a low dose of actinomycin D (ActD) or by siRNA-mediated silencing of its large catalytic subunit, RPA194 [29,30]. Localization of CRM1 to the CB and its relocation to the nucleolus are abrogated when cells are treated with LMB [24,29,30], suggesting that localization to both intranuclear compartments requires NES binding activity. However, it has not yet been tested how CRM1 intranuclear localization is affected by mutations that impact NES binding and nuclear export activity. We hypothesized that these mutants could shed further light on how localization of CRM1 inside the nucleus is modulated.

**Figure 1:**
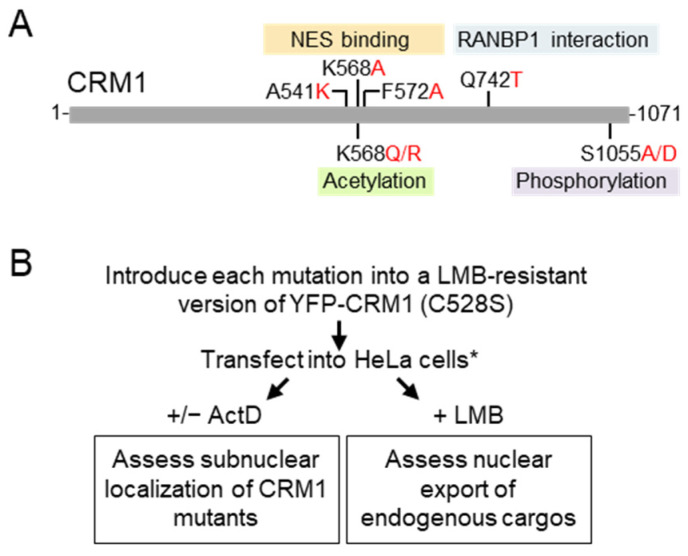
CRM1 residues mutated in this study and experimental design. (**A**) Schematic representation of human CRM1 protein showing the position of the key residues and their mutations analyzed in this study. The aspects of CRM1 function and regulation where these residues are reportedly involved (NES binding, RanBP1 binding, acetylation, phosphorylation) are also indicated. (**B**) Workflow diagram illustrating the experimental design of the study. (*) Some experiments were also carried out in HEK293T cells.

In this study, we aim to investigate how mutations targeting key functional residues in CRM1 (Figure 1A) affect the intranuclear localization of the receptor, as well as to confirm, extend, and independently validate the effect of these mutations on the nuclear export activity of CRM1. To this end, as summarized in Figure 1B, we introduced each of these mutations into an LMB-resistant (C528S) version of YFP-CRM1 (referred to as YFP-CRM1*) and assessed their intranuclear localization and their ability to promote the nuclear export of well-established endogenous CRM1 cargos.

## 2. Materials and Methods

### 2.1. Plasmids and Site-Directed Mutagenesis

The plasmid encoding wild-type YFP-CRM1 has been previously described [31]. LMB-resistant YFP-CRM1* was generated by introducing the C528S mutation into YFP-CRM1 using the Quick-Change Lightning Site-Directed Mutagenesis Kit (Agilent Technologies, Santa Clara, CA, USA) according to the manufacturer’s instructions. Subsequently, the same kit was used to introduce the A541K, F572A, K568A, K568Q, K568R, S1055A, S1055D, and Q742T mutations into YFP-CRM1*. To confirm the presence of the intended mutation and the absence of any unwanted sequence error, all the new constructs were analyzed by DNA sequencing (StabVida, Caparica, Portugal).

### 2.2. Cell Culture, Plasmid Transfection, and Drug Treatments

Human cervical carcinoma HeLa cells and human embryonic kidney 293T cells (HEK293T) were obtained from ECACC (Salisbury, UK). Cells were grown in Dulbecco’s modified Eagle’s medium (DMEM) supplemented with 10% fetal bovine serum (FBS), 100 U/mL penicillin, and 100 μg/mL streptomycin (all from Gibco (ThermoFisher Scientific), Waltham, MA, USA) at 37 °C in a humidified atmosphere containing 5% CO_2_. 24 h before transfection, cells were seeded in 12-well plates. A glass coverslip was placed in each well before seeding the cells. Plasmid transfections were carried out using X-tremeGENE 9 DNA transfection reagent (Roche Diagnostics, Basel, Switzerland) following manufacturer’s instructions. The CRM1 inhibitor Leptomycin B (LMB, Apollo Scientific, Bredbury, Stockport, UK) was used at a final concentration of 6 ng/mL for 3 h. Actinomycin D (ActD, Sigma-Aldrich, Saint Louis, MO, USA) was used at a final concentration of 100 ng/mL for 3 h. TNFα (R&D Systems, Minneapolis, MN, USA) was used at a final concentration of 10 ng/mL for 30 min.

### 2.3. Immunofluorescence and Microscopy Analysis

Cells were fixed with 3.7% formaldehyde in PBS for 30 min, permeabilized with 0.2% Triton X-100 in PBS for 10 min, blocked for 1 h in blocking solution (3% BSA in PBS), and incubated for 1 h at room temperature with primary antibodies diluted in blocking solution. The following primary antibodies and dilutions were used: rabbit anti-CRM1 (Novus NB100-79802, 1:300), rabbit anti-coilin (Proteintech 10967-1-AP, 1:800), rabbit anti-NMD3 (Proteintech 16060-1-AP, 1:300), rabbit anti-RanBP1 (Proteintech 27804-1-AP, 1:200), mouse anti-SQSTM1 (Santa Cruz sc-28359, 1:200), and rabbit anti-NFκB p65 (Santa Cruz sc-372, 1:300). As indicated in the Results Section, a second anti-NFκB p65 antibody (8242P from Cell Signaling Technology, Danvers, MA, USA) was used to confirm some of the results with this protein. After incubation with the primary antibody, cells were washed three times with PBS and incubated with anti-mouse (Invitrogen, Carlsbad, CA, USA, A11032) or anti-rabbit (Invitrogen A11012 or A32740) Alexa Fluor 594-conjugated secondary antibodies, diluted 1:400 in blocking solution for 1 h at room temperature. Coverslips were finally washed with PBS and mounted onto microscope slides using Vectashield mounting medium with 4′,6-diamidino-2-phenylindole (DAPI) (Vector Laboratories, Burlingame, CA, USA).

Samples were examined using either a Zeiss Axioskop fluorescence microscope or a Zeiss LSM 880 Airyscan confocal microscope and images were captured with NIS Elements (version 3.0) or ZEN Black software (version 2.3), respectively. Using these images, we determined the percentage of transfected cells with Cajal body or nucleolar localization of the different YFP-CRM1* variants. On the other hand, the micrographs were subjected to image analysis to quantify the intensity of fluorescent signals, as described below.

### 2.4. Image Analysis

Image analysis was carried out manually using Fiji software (version 2.14.0) [32]. To establish the CB/nucleoplasm and nucleolar/nucleoplasm ratios, the intensity of the YFP-CRM1* signal was quantified in one CB or nucleolus per cell, as well as in the nucleoplasm of the same cell. Quantitative analysis of RanBP1 and p65 localization was carried out using two slightly different approaches. In HeLa cells, which have a relatively large cytoplasm, the mean intensity of the immunofluorescent signal in the nucleus and the cytoplasm was quantified and the nuclear to cytoplasm (N/C) ratio was calculated for each cell. In HEK293T cells, which have a smaller cytoplasm and tend to grow in clusters, we found it more difficult to reliably quantify the fluorescence intensity in the cytoplasm of individual cells. Thus, we quantified the average intensity of RanBP1 or p65 fluorescence in the whole micrograph (usually containing more than 100 cells) and used this value as the “total” fluorescence. Then, the mean intensity of the fluorescent signal in the nucleus of individual cells was quantified and a nuclear/total ratio was calculated for each cell. The mean intensity of the YFP signal in the nucleus of the cells was also quantified. The number of cells examined in each experiment is indicated in the corresponding figure legend.

### 2.5. Statistical Analysis

GraphPad Prism version 7 software was used to generate graphs and to carry out statistical analysis. Statistical comparisons between samples were performed using Student’s *t* test, and differences were considered statistically significant when *p* < 0.05.

## 3. Results

### 3.1. An YFP-Tagged, LMB Resistant Version of CRM1 Recapitulates the Intranuclear Localization of Endogenous CRM1 in HeLa Cells

**Figure 2:**
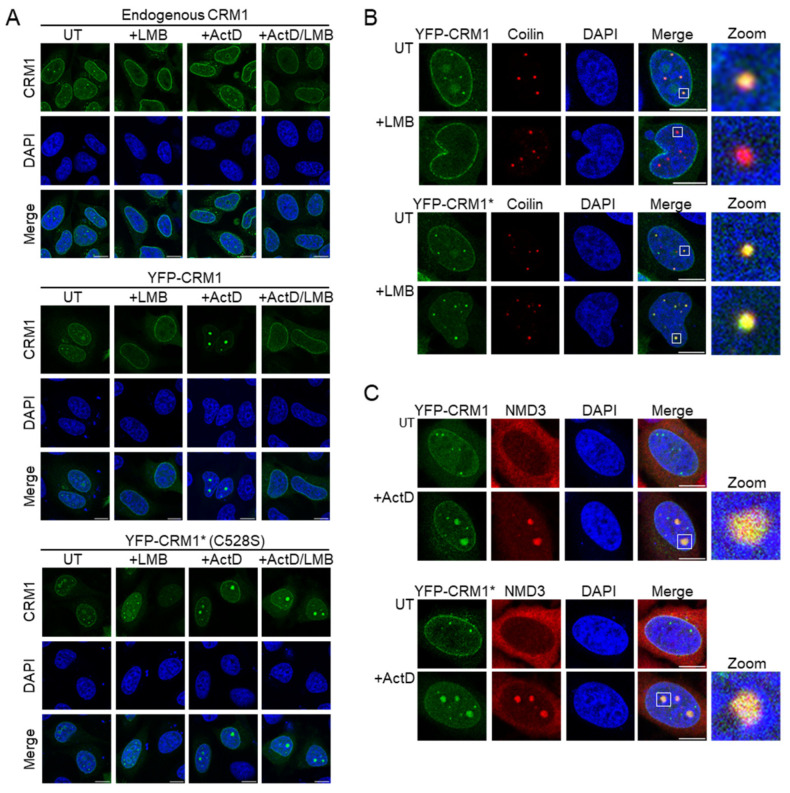
An LMB resistant, YFP-tagged version of CRM1 recapitulates the intranuclear localization of endogenous CRM1 in HeLa cells. (**A**) Confocal images showing representative examples of the localization of endogenous CRM1 (upper set of panels), YFP-CRM1 (middle set of panels) and LMB-resistant YFP-CRM1* (lower set of panels) in HeLa cells untreated (UT) or treated with the indicated drugs for 3 h. LMB was used at 6 ng/mL and ActD at 100 ng/mL. (**B**) Confocal images showing representative examples of the co-localization of YFP-CRM1 and YFP-CRM1* with the Cajal body (CB) marker coilin in the nucleus of HeLa cells untreated (UT) or treated with LMB. Zoom images show magnification of one selected CB (white square). (**C**) Confocal images showing representative examples of the localization of YFP-CRM1, YFP-CRM1*, and endogenous NMD3 in the nucleus of HeLa cells. YFP-CRM1 and YFP-CRM1* co-localize with NMD3 in the nucleoli of ActD-treated cells but not in untreated (UT) cells. Zoom images show magnification of one selected nucleolus (white square). In all the panels DAPI was used to stain the nuclei, and the scale bar represents 10 μm.

Endogenous CRM1 has been shown to localize to the Cajal body (CB) in untreated HeLa cells [23,24] and to relocate to the nucleolus when the cells are treated with a low concentration (50–100 ng/mL) of actinomycin D, which inhibits RNA polymerase I [29,30]. Targeting to both intranuclear compartments was abrogated when the cells were treated with LMB. Using immunofluorescence with a polyclonal anti-CRM1 antibody, we confirmed these observations in HeLa cells (Figure 2A, upper panels). We also examined the localization of endogenous CRM1 in HEK293T, another cell line widely used in cell biology studies. Unlike in HeLa cells, CRM1 did not localize in CB-resembling nuclear foci in untreated HEK293T cells, although it readily accumulated in the nucleolus upon ActD treatment (Supplementary Figure S1A). Of note, the presence of CBs in both cell lines was confirmed using coilin as a marker [33] (Supplementary Figure S1B), although the fact that both anti-CRM1 and anti-coilin antibodies are raised in rabbit precluded simultaneous detection of both proteins by co-immunofluorescence. The absence of endogenous CRM1 from the CBs of HEK293T cells might reflect differences in the expression of the protein(s) that mediate CRM1 location to this structure in the different cell lines.

Next, we examined the intranuclear localization of YFP-tagged CRM1 in transiently transfected HeLa cells. It should be pointed out that throughout the present study, we focus our analyses on transfected cells expressing low to moderate levels of the ectopic protein. In line with a previous report [34], we found that YFP-CRM1 localizes to CBs in untreated, but not in LMB-treated HeLa cells (Figure 2A, middle panels). Furthermore, we found that YFP-CRM1 relocates to the nucleolus upon ActD treatment, but not when the cells are simultaneously treated with ActD and LMB. Finally, we carried out similar experiments with an LMB-resistant version of YFP-CRM1 bearing the C528S mutation (hereafter referred to as YFP-CRM1*), which should localize properly, even in the presence of the inhibitor.

When transfected into HeLa cells, YFP-CRM1* mimicked the intranuclear localization of endogenous CRM1 and YFP-CRM1 (Figure 2A, lower panels). As expected, however, the CB localization and nucleolar relocation of YFP-CRM1* were not disrupted by LMB treatment. Finally, to further demonstrate the localization of YFP-CRM1 and YFP-CRM1* to these intranuclear compartments, cells were co-stained for the CB marker coilin [33] (Figure 2B) and for NMD3 (Figure 2C), which localizes to the nucleoli in ActD-treated cells [30]. Additional experiments with a second nucleolar marker, p14 ^ARF^ [35], were also performed (Supplementary Figure S2A).

In summary, we conclude that the localization of ectopically expressed YFP-CRM1* faithfully reproduces the localization of the endogenous CRM1 protein in HeLa cells. In the rest of the experiments described in this report, YFP-CRM1* is used as the “wild type” protein into which the mutations to be tested will be introduced.

### 3.2. CRM1 Mutations Differently Alter the Intranuclear Localization of the Receptor

HeLa cells were next transfected with YFP-CRM1* plasmids containing each of the mutations indicated in Figure 1A to determine how these sequence changes affect the intranuclear localization of CRM1.

**Figure 3:**
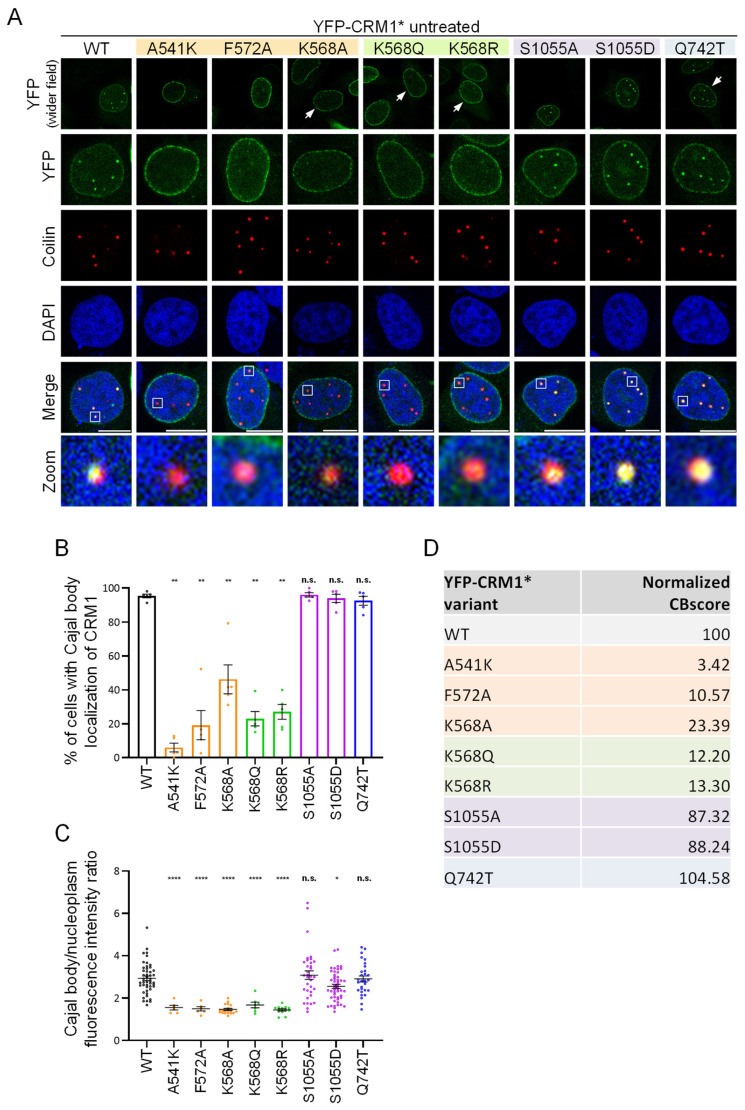
CRM1 mutations differently alter the localization of the receptor to the Cajal body in HeLa cells. (**A**) Confocal images showing representative examples of the co-localization of the different YFP-CRM1* mutants with the CB marker coilin in the nucleus of untreated HeLa cells. “Wider field” panels are included to show a larger area of the cell cytoplasm. Arrowheads indicate the nucleus depicted in more detail in the panels below. Zoom images show magnification of one selected CB (white square). DAPI was used to stain the nuclei, and the scale bar represents 10 μm. (**B**) Graph showing the percentage of HeLa cells with CB localization of each YFP-CRM1* variant. Bars represent the mean of five independent experiments and error bars indicate standard deviation (SD). Student’s *t* test was used to compare each mutant to the WT. n.s.: non-significant; (**) *p* < 0.01. (**C**) Results of a representative experiment where the fluorescence intensity of each YFP-CRM1* variant at the CB and the nucleoplasm was quantified by image analysis using Fiji. The graph shows the CB/nucleoplasm intensity ratio. Each dot represents a single cell and the mean (+/− SD) is also shown. Student’s *t* test was used to compare each mutant to the WT. n.s.: non-significant; (*) *p* < 0.05; (****) *p* < 0.0001. (**D**) Table summarizing the normalized Cajal body score (Normalized CBscore) for each variant. This score was calculated from five independent experiments (at least 25 cells per condition were scored in each experiment), by multiplying the mean percentage of cells with CB localization by the mean CB/nucleoplasm fluorescence intensity ratio (see Supplementary Table S1). The score of each mutant was normalized to the score of wild type YFP-CRM1*, set at 100.

Figure 3A shows examples of the localization of these mutants in the nucleus of untreated cells. Coilin was used as a CB marker in these experiments. To semi-quantitatively assess the effect of each mutation, the percentage of transfected cells showing CB localization of YFP-CRM1* was determined (Figure 3B), and the ratio between the intensity of the fluorescent signal in the CB and in the nucleoplasm was analyzed using Fiji (Figure 3C). From these measurements (obtained in 5 independent experiments), we derived a metric that we called “Cajal body localization score” (CBscore) (Figure 3D and Supplemmentary Table S1). In order to facilitate comparisons, the score of the different mutants was normalized to the CBscore of wild type YFP-CRM1* (set at 100). The three NES binding groove mutations severely impaired CB localization, albeit to a different extent. Thus, localization to this compartment was almost fully abrogated by the A541K mutation (normalized CBscore 3.42), but partially retained in the case of the F572A and K568A mutants (normalized CBscore 10.57 and 23.39, respectively). Regarding the PTM-related mutants, both K568Q (acetylation-mimicking) and K568R (non-acetylatable) variants showed severely impaired CB localization (normalized CBscore 12.20 and 13.30, respectively), while S1055A (non-phosphorylatable) and S1055D (phosphomimetic) mutants showed only a minor reduction (normalized CBscore 87.32 and 88.24, respectively). Finally, the Q742T mutant protein was fully able to localize to the CB (normalized CBscore 104.58). Of note, a subset of the mutations (A541K, F572A, K568A, S1055A, and S1055D) were also introduced into YFP-CRM1 (not bearing the C528S mutation) and tested to ensure that the presence of the LMB-resistant mutation was not noticeably affecting the localization of these CRM1 proteins (Supplementary Figure S3).

**Figure 4:**
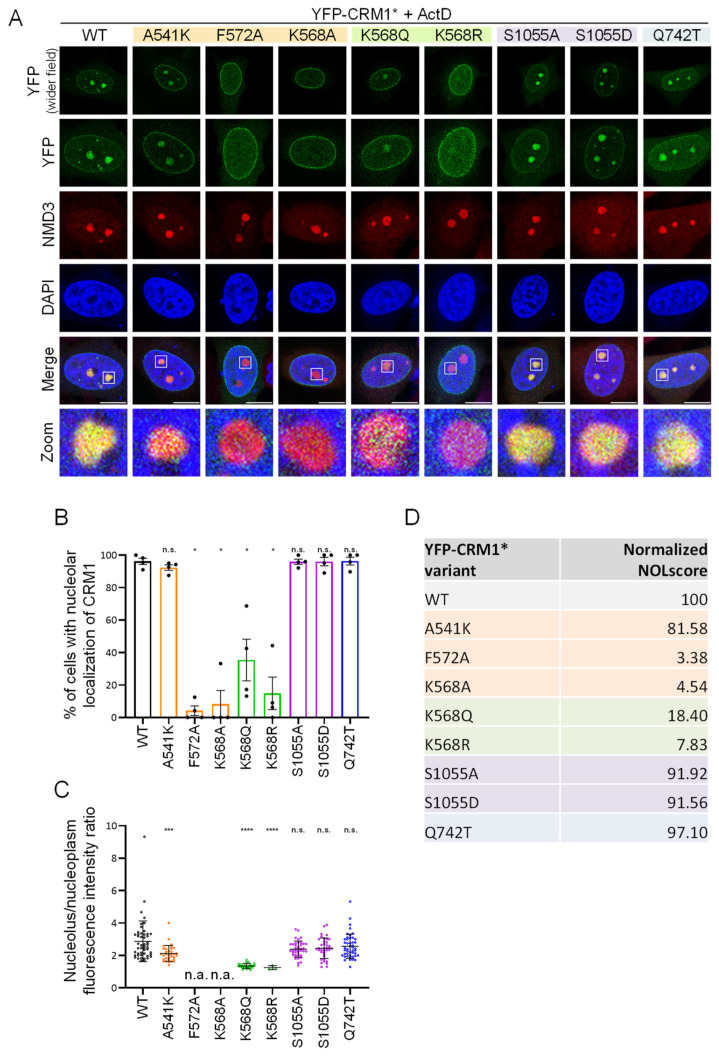
CRM1 mutations differently alter the relocation of the receptor to the nucleolus in actinomycin D-treated HeLa cells. (**A**) Confocal images showing representative examples of the co-localization of the different YFP-CRM1* mutants with endogenous NMD3 in the nucleus of HeLa cells treated with ActD (100 ng/mL for 3 h). “Wider field” panels are included to show a larger area of the cell cytoplasm. Zoom images show magnification of one selected nucleolus (white square). DAPI was used to stain the nuclei, and the scale bar represents 10 μm. (**B**) Graph showing the percentage of HeLa cells with nucleolar localization of each YFP-CRM1* variant. Bars represent the mean of four independent experiments and error bars indicate standard deviation (SD). Student’s *t* test was used to compare each mutant to the WT. n.s.: non-significant; (*) *p* < 0.05. (**C**) Results of a representative experiment where the fluorescence intensity of each YFP-CRM1* variant at the nucleolus and the nucleoplasm was quantified by image analysis using Fiji. The graph shows the nucleolus/nucleoplasm intensity ratio. Each dot represents a single cell and the mean (+/− SD) is also shown. Student’s *t* test was used to compare each mutant to the WT. n.a: not assessed; n.s.: non-significant; (***) *p* < 0.001; (****) *p* < 0.0001. (**D**) Table summarizing the normalized nucleolar relocation score (Normalized NOLscore) for each variant. This score was calculated from four independent experiments (at least 25 cells per condition were scored in each experiment) by multiplying the mean percentage of cells with nucleolar localization by the mean nucleolar/nucleoplasm fluorescence intensity ratio (see Supplementary Table S1). The score of each mutant was normalized to the score of wild type YFP-CRM1*, set at 100.

We also examined the localization of these mutants in HeLa cells treated with a low concentration of ActD (100 ng/mL for 3 h). Representative examples of the results are shown in Figure 4A. In these experiments, NMD3 (Figure 4) or p14 ^ARF^ (Supplementary Figure S2B) were used as markers of the nucleolus. As above, a semi-quantitative assessment was carried out by determining the percentage of transfected cells with nucleolar localization of YFP-CRM1* (Figure 4B) and measuring the ratio between the intensity of the fluorescent signal in the nucleolus and in the nucleoplasm (Figure 4C). A metric termed “nucleolar relocation score” (NOLscore) (Figure 4D and Supplementary Table S1) was derived from these measurements (in four independent experiments) and normalized to the NOLscore of wild type YFP-CRM1* (set at 100). Remarkably, the NES binding groove mutants showed a strikingly different ability to undergo nucleolar relocation. Thus, while the A541K mutant was only slightly impaired (normalized NOLscore 81.58), relocation was fully abrogated in the case of the F572A and K568A mutants (normalized NOLscore 3.38 and 4.54, respectively). On the other hand, the effect of PTM-related mutations on nucleolar relocation was similar to their effect on CB localization. Thus, relocation was severely impaired in the case of K568Q and K568R mutants (normalized NOLscore 18.40 and 7.83, respectively), but essentially unaffected in the case of S1055A and S1055D (normalized NOLscore 91.92 and 91.56, respectively). Finally, the Q742T mutant readily relocated to the nucleolus (normalized NOLscore 97.10). To further substantiate our findings, we also assessed the intranuclear localization of the mutants in ActD-treated HEK293T cells. The results of this experiment (Supplementary Figure S4) were similar to those in HeLa cells, thus confirming that the effect of these mutations is not cell line-specific.

In summary, these results show that CRM1 does not require S1055 phosphorylation to achieve its proper intranuclear localization and reveal surprisingly divergent effects of different NES binding grove mutations.

### 3.3. Selection of Endogenous Cargos to Evaluate the Nuclear Export Activity of CRM1 Mutants

The second aim of our study was to confirm, extend, and independently validate previous findings regarding the effect of the mutations on the nuclear export activity of CRM1. To this end, our approach was to determine the nucleocytoplasmic localization of endogenous CRM1 cargos in cells transfected with YFP-CRM1* plasmids encoding the different mutants. 16 h after transfection, cells are treated for 3h with LMB, fixed, and immunostained for each cargo. Since the activity of endogenous CRM1 is blocked by LMB, the nucleocytoplasmic distribution of the cargo in this experimental setting depends solely on the export activity of the ectopically expressed YFP-CRM1* mutant protein.

**Figure 5:**
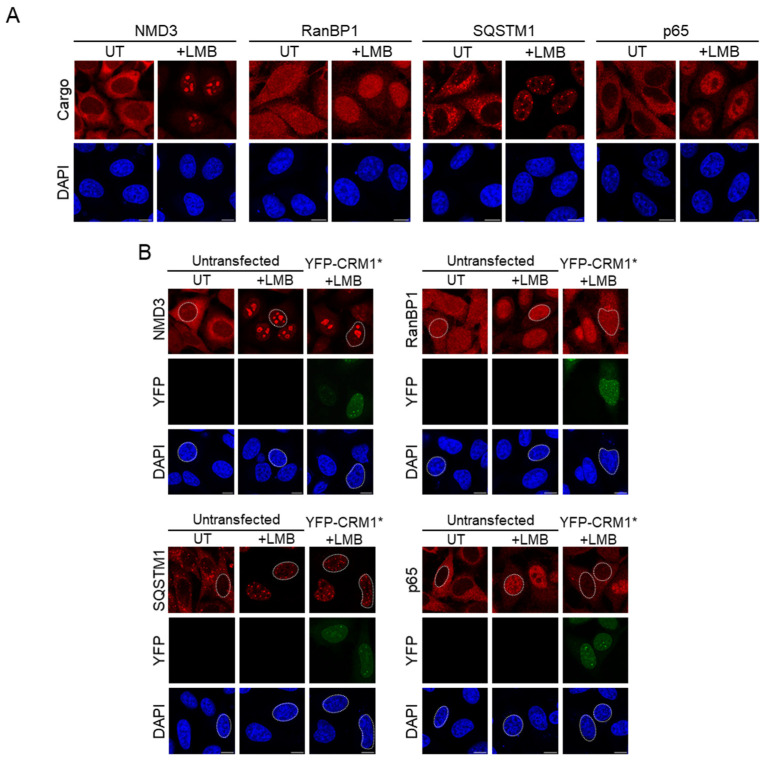
Selection of endogenous cargos as markers to evaluate the nuclear export activity of CRM1 mutants. (**A**) Confocal images showing representative examples of the localization of four endogenous CRM1 cargos (NMD3, RanBP1, SQSTM1, and p65) in HeLa cells untreated (UT) or treated with LMB (6 ng/mL for 3 h). (**B**) Confocal images showing representative examples of the localization of these cargos in untransfected HeLa cells untreated (UT) or treated with LMB, as well as in LMB-treated transfected cells expressing low to moderate levels of YFP-CRM1*. DAPI was used to stain the nuclei, and the scale bar represents 10 μm.

As a first step, we choose four known CRM1 cargo proteins that have been previously reported to localize to the cytoplasm of untreated cells and relocate to the nucleus when the cells are treated with LMB: NMD3 [30,36,37], RanBP1 [38,39], SQSTM1 [40], and the p65 subunit of NFκB [41,42]. As expected, LMB treatment induced an evident nuclear accumulation of the four proteins (Figure 5A). In LMB-treated HeLa cells, RanBP1, and p65 were mainly localized to the nucleoplasm, while SQSTM1 localized to small nuclear speckles (most likely PML bodies, as previously described [40]), and NMD3 prominently accumulated in nucleoli. Of note, previous studies using ectopically expressed NMD3 have reported conflicting observations regarding the extent of NMD3 nucleolar accumulation caused by LMB treatment [30,36,37]. In our analysis of endogenous NMD3, a faint nucleolar signal was noticeable in about 5% of untreated HeLa cells, and a strong nucleolar accumulation was noted in nearly 100% of LMB-treated cells, in line with the findings of Trotta et al. using GFP-tagged NMD3 [37].

Next, we tested the ability of YFP-CRM1* to revert the LMB-induced nuclear accumulation of these cargos. As shown in Figure 5B, expression of YFP-CRM1* at low to moderate levels clearly reduced the amount of RanBP1 and p65 in the nucleus of LMB-treated cells. However, at similar levels of expression, YFP-CRM1* failed to consistently reduce the nuclear localization of SQSTM1 and NMD3. These observations suggest that nuclear RanBP1 and p65 are readily available for CRM1-mediated export in LMB-treated HeLa cells, while export of SQSTM1 and NMD3 may be hampered by retention in the nucleus, perhaps due to their anchoring to the subnuclear structures described above.

In order to further explore the effect that nuclear retention may have on the behavior of CRM1 cargos, we focused on p65. Although this protein (usually in a dimer with p50, another NFκB family member) is mostly localized in the cytoplasm of untreated cells, it undergoes constant nucleocytoplasmic shuttling. Nuclear accumulation of p65 in LMB-treated cells is due to export blockade, but p65 also accumulates in the nucleus when cells are treated with cytokines that activate the NFκB pathway, such as TNFα. Upon pathway activation, however, nuclear p65 binds to specific DNA sequences and undergoes posttranslational modifications that may enhance its retention in the nucleus, reviewed in [43,44]. We compared the ability of YFP-CRM1* to export p65 when its nuclear accumulation is induced by LMB or by TNFα. Strikingly, we found that YFP-CRM1* was completely unable to export p65 when its nuclear accumulation is induced by TNFα (Supplementary Figure S5), clearly illustrating how nuclear retention may effectively prevent CRM1-mediated nuclear export of cargos.

Altogether, these results suggest that not all bona fide CRM1 cargos are suitable markers to assess the nuclear export function of CRM1 in our experimental setting and they highlight the importance of selecting both optimal markers and experimental conditions to carry out these analyses. Here, we selected RanBP1 and p65 to compare the ability of the different CRM1 mutants to mediate cargo export.

### 3.4. CRM1 Mutations Differently Alter Nuclear Export of RanBP1 and p65

**Figure 6:**
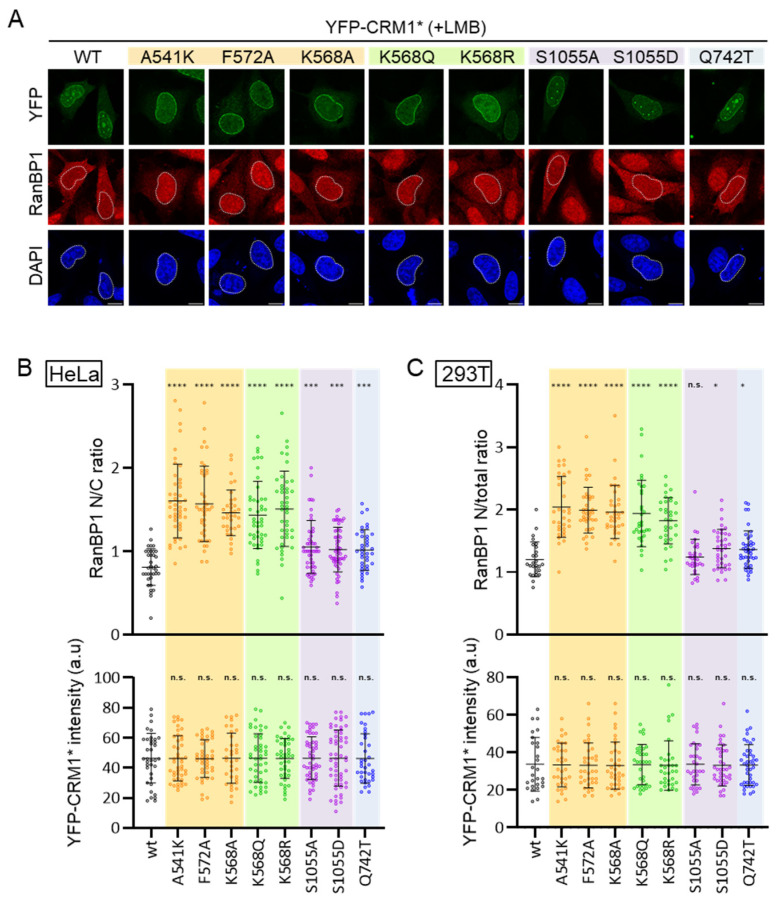
CRM1 mutations differently alter nuclear export of RanBP1. (**A**) Confocal images showing representative examples of the localization of endogenous RanBP1 in LMB-treated HeLa cells expressing low to moderate levels of each YFP-CRM1* variant. DAPI was used to stain the nuclei, and the scale bar represents 10 μm. (**B**) Graphs showing the nuclear to cytoplasmic (N/C) ratio of RanBP1 (upper section) and the intensity of the YFP fluorescence (lower section) in HeLa cells expressing the indicated YFP-CRM1* mutant. (**C**) Graphs showing the nuclear to total (N/total) ratio of RanBP1 (upper section) and the intensity of the YFP fluorescence (lower section) in HEK293T cells expressing the indicated YFP-CRM1* mutant. In B and C panels, each dot represents a single cell where both RanBP1 and YFP-CRM1* fluorescence intensity were determined by image analysis using Fiji. The horizontal lines indicate the mean, and error bars represent SD. Student’s *t* test was used to compare each mutant to the WT. n.s.: non-significant; (*) *p* < 0.05; (***) *p* < 0.001; (****) *p* < 0.0001.

The localization of endogenous RanBP1 was determined by immunofluorescence in LMB-treated HeLa cells expressing the different YFP-CRM1* mutants. Representative examples are shown in Figure 6A. Image analysis was used to quantify the intensity of the RanBP1 signal in the nucleus and the cytoplasm, and thus determine the nuclear/cytoplasmic (N/C) ratio. Similar experiments were carried out in HEK293T cells and image analysis was used to derive the nuclear/total (N/total) ratio of RanBP1 fluorescence, as detailed in the Materials and Methods Section. In both HeLa and HEK293T experiments, the intensity of the nuclear YFP signal was also determined in each cell scored for RanBP1 localization. In our view, this is a crucial control to ensure similar expression levels of the different CRM1 mutants and thus allow for a meaningful comparison of their export activity.

Each CRM1 mutant was tested in at least three independent experiments in both HeLa and HEK293T cells, and at least 25 transfected cells were scored per experiment. The results of one representative experiment in each cell line are shown in Figure 6B,C and a summary of the results of the different experiments is provided as Supplementary Table S2. The three NES binding groove mutations (A541K, F572A, and K568A), as well as the two acetylation-related mutations (K568Q and K568R), severely disrupted RanBP1 export in both cell lines. In marked contrast, the phosphorylation-related mutations (S1055A and S1055D), as well as the Q742T mutation, only slightly reduced the ability of CRM1 to promote nuclear export of RanBP1. This was evident in HeLa cells and was even clearer in HEK293T cells. In fact, in the experiment shown in Figure 6C, the differences in RanBP1 export between these mutants and the wild type did not reach (S1055A) or barely reached (S1055D and Q742T) statistical significance in this cell line.

**Figure 7:**
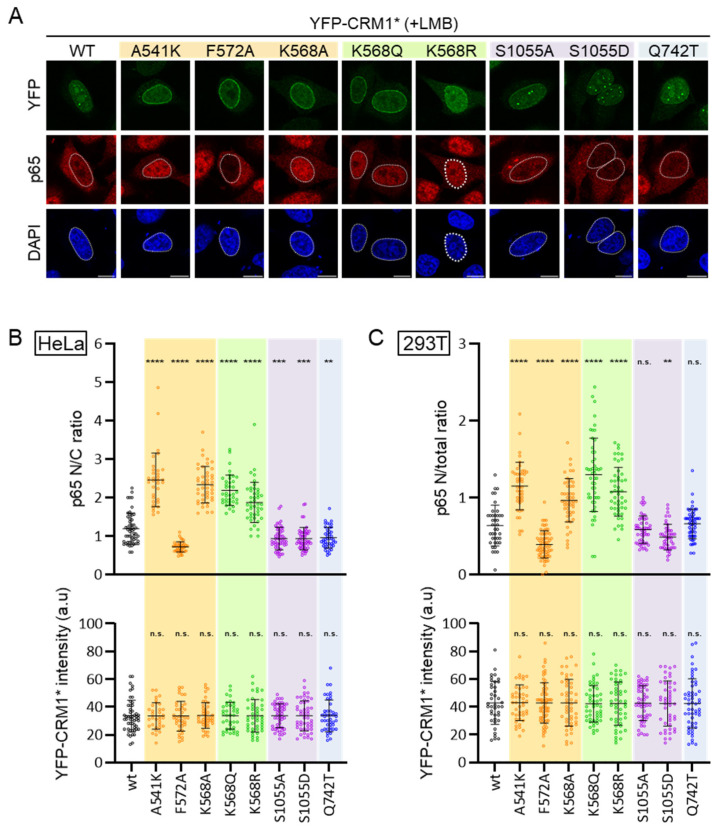
CRM1 mutations differently alter the nuclear export of p65. (**A**) Confocal images showing representative examples of the localization of endogenous p65 in LMB-treated HeLa cells expressing low to moderate levels of each YFP-CRM1* variant. DAPI was used to stain the nuclei, and the scale bar represents 10 μm. (**B**) Graphs showing the nuclear to cytoplasmic (N/C) ratio of p65 (upper section) and the intensity of the YFP fluorescence (lower section) in HeLa cells expressing the indicated YFP-CRM1* mutant. (**C**) Graphs showing the nuclear to total (N/total) ratio of p65 (upper section) and the intensity of the YFP fluorescence (lower section) in HEK293T cells expressing the indicated YFP-CRM1* mutant. In B and C panels, each dot represents a single cell where both p65 and YFP-CRM1* fluorescence intensity were determined by image analysis using Fiji. The horizontal lines indicate the mean, and error bars represent SD. Student’s *t* test was used to compare each mutant to the WT. n.s.: non-significant; (**) *p* < 0.01; (***) *p* < 0.001; (****) *p* < 0.0001.

A similar set of experiments was carried out using p65 as cargo. Figure 7A shows representative examples of the localization of endogenous p65 in LMB-treated HeLa cells expressing the different YFP-CRM1* mutants. Image analysis and quantification of p65 N/C ratio (in HeLa) or N/total ratio (in HEK293T) were performed as described above for RanBP1. Each CRM1 mutant was tested in at least three independent experiments in both HeLa and HEK293T cells, and at least 25 transfected cells were scored per experiment. The results of one representative experiment in each cell line are shown in Figure 7B,C, and a summary of the results of the different experiments is provided as Supplementary Table S2. The ability of most mutants to mediate p65 export closely paralleled their ability to mediate RanBP1 export. Thus, A541K, K568A, K568Q, and K568R mutants had severely impaired p65 export, while S1055A, S1055D, and Q742T mutants showed minor or no differences with respect to the wild type. Unexpectedly, the NES-binding groove mutation F572A, which fully abrogates RanBP1 export (see above), clearly increased the ability of CRM1 to mediate p65 export in both HeLa and HEK293T cells. Of note, essentially identical results were obtained using a different p65 antibody (Cell Signaling Technology #8242), thus confirming the reproducibility of these observations.

These results clearly show that the ability of CRM1 to mediate nuclear export of RanBP1 and p65 is essentially unaffected by two mutations previously reported to abrogate its function: S1055A [20] and Q742T [17]. Furthermore, we have identified a striking example of a NES-binding groove mutation (F572A) that either reduces or enhances export of specific cargos.

## 4. Discussion

Mutational analysis has been instrumental to characterize the nuclear export activity of CRM1 and has pinpointed several key functional residues in this protein. In the present study, we investigate how the mutation of these residues may provide information on another important aspect of CRM1 biology: its dynamic localization to two specific intranuclear compartments: the Cajal body (CB) and the nucleolus. In addition, we further investigate how these mutations affect the nuclear export of endogenous cargos.

Previous studies indicate that the localization of CRM1 to the CB and nucleolus in HeLa cells requires NES binding, as both are disrupted by LMB [24,29,30], an inhibitor that specifically blocks CRM1/NES interactions. Here, we provide direct evidence further supporting these observations, by showing that a mutation that confers LMB resistance (C528S) restores the proper intranuclear localization of CRM1 in the presence of the inhibitor. Furthermore, we identify a NES binding groove mutation (A541K) that severely disrupts CB localization, but not nucleolar relocation. Therefore, the A541K represents a separation-of-function mutation that reveals the independent nature of these two aspects of CRM1 intranuclear localization. This view is further supported by our observation that endogenous CRM1 does not localize to the CB in HEK293T cells, but clearly relocates to the nucleoli when these cells are treated with ActD. These findings suggest that the localization of CRM1 to CB and nucleolus may be mediated by its binding to different NES-bearing cargo proteins. In this regard, the silencing of NMD3 has been shown to prevent the relocation of CRM1 to the nucleolus in ActD-treated HeLa cells [30]. However, while expression of NMD3 may be necessary for the nucleolar relocation of CRM1, our findings in LMB-treated cells (see Figure 2 and Figure 5) clearly show that a strong accumulation of endogenous NMD3 in the nucleolus is not sufficient to relocate endogenous or ectopic CRM1 to this compartment. This suggests a more complex interplay between these proteins in regulating each other’s localization, which should be further characterized in future studies.

We also tested the possibility that PTMs modulate the intranuclear localization of CRM1 by introducing mutations into a yet uncharacterized potential acetylation site (K568) as well as the previously characterized S1055 phosphorylation site [20]. We generated CRM1 mutants bearing modification-mimicking (K > Q and S > D) or non-modifiable (K > R and S > A) residues at these sites. Acetylation of CRM1 K568 residue has been reported in multiple global proteomics studies according to the PhosphoSitePlus repository. However, mutations mimicking or preventing acetylation of this site did not provide conclusive information in our assays, since both K568Q and K568A mutants were severely impaired in their ability to localize to CB and nucleolus. On the other hand, both phosphorylation mimicking (S1055D) and non-phosphorylatable (S1055A) mutants readily localized to both compartments, indicating that S1055 phosphorylation does not play a significant role regulating the intranuclear localization of CRM1. This was unexpected, given that STK38-mediated phosphorylation of this residue has been previously claimed to be crucial for the nuclear export activity of CRM1 [21]. Similarly surprising was our finding that the Q742T mutation, previously reported to abrogate cargo export [17], did not have any deleterious effect on CRM1 intranuclear localization. In an attempt to further clarify these observations, we aimed to confirm and extend previous findings regarding the effect that our panel of mutations has on the “canonical” activity of CRM1 as a nuclear export receptor.

We began by selecting four previously described endogenous cargos of CRM1: NMD3, RanBP1, SQSTM1, and p65, and testing their suitability as markers to evaluate the nuclear export activity of the different CRM1 mutants. As expected, the four proteins relocated from the cytoplasm to the nucleus when cells were treated with LMB. LMB-induced nuclear accumulation of RanBP1 and p65 was readily reverted by ectopic expression of LMB-resistant YFP-CRM1*. In contrast, the re-export of NMD3 and SQSTM1 by YFP-CRM1* appears to be hindered in LMB-treated cells, which may be due, at least in part, to nuclear retention of these proteins at the nucleolus and PML bodies, respectively. We conclude that these two cargos are not well suited as markers for nuclear export activity and thus, we decided to use RanBP1 and p65 as markers in subsequent experiments. Interestingly, these observations also show that our experimental setting can be useful to explore the dynamic behavior and potential nuclear retention of other CRM1 cargos that relocate to the nucleus upon different stimuli. This is illustrated by comparing the effect of YFP-CRM1* expression on the localization of p65 in cells treated with either LMB or TNFα. Both drugs induce a seemingly similar accumulation of p65 in the nucleus. However, YFP-CRM1* mediated re-export of p65 is severely hampered in TNFα-treated cells, consistent with the engagement of previously described mechanisms of p65 retention in the nucleus [43,44].

After selecting RanBP1 and p65 as optimal markers, we evaluated to what extent the different CRM1 mutants were able to mediate their re-export in LMB-treated cells. We carried out these experiments in both HeLa and HEK293T cells, with nearly identical results, showing that our findings are not limited to a specific cell line.

The different mutants demonstrate considerable diversity in their nuclear export activity. In line with previous findings [9,10,14,15], NES binding groove mutations A541K and K568A severely impaired export of both RanBP1 and p65. Surprisingly, we observed a striking cargo-specific effect in the case of the F572A mutant: it was defective in RanBP1 export, but it mediated nuclear export of p65 even more efficiently than wild type CRM1. This result was unanticipated, since we have previously shown that the F572A mutation consistently reduced nuclear export activity when tested against a panel of reporters containing 14 different NES motifs [15]. We speculate that, while being unable to efficiently bind most NESs, the F572A mutant might be particularly efficient in binding and exporting a specific subset of NES motifs. In this regard, it remains to be established whether the export-enhancing effect of F572A is limited to p65 or it extends to additional cargos. Dissecting the molecular underpinnings behind enhanced p65 export by CRM1 F572A will require future experiments and may be challenging, as nuclear export of p65 is notoriously complex, being regulated by several independent NES motifs in p65 itself and in IκBα and by its dimerization partner p50 [41,42,45].

Acetylation is a common regulatory PTM for many cellular proteins, reviewed in [46], but little is known about its potential role in regulating CRM1 nuclear export activity. The location of the K568 potential acetylation site in the NES-binding groove, and the key role of this residue in NES motif binding and export [15,16] led us to hypothesize that K568 acetylation might modulate CRM1 export activity. However, both acetylation mimicking (K568Q) and acetylation preventing (K568R) mutations abrogated the export of RanBP1 and p65, mirroring the deleterious effect of these mutations on the intranuclear localization of CRM1. Although it is known to have some limitations [47], the mutagenesis strategy used in our study is widely used in the field of protein acetylation. This approach, however, did not allow us to draw any clear conclusion regarding a potential role of K568 acetylation on CRM1 localization or function. More sophisticated methods, such as genetic codon expansion technologies [48], may be applied in the future to address this issue.

Phosphorylation of CRM1 at S1055 by the STK38 kinase has been shown to regulate nuclear export of several autophagy-related proteins [20]. It has been proposed that, by allosterically releasing the autoinhibited conformation of the receptor, this modification may be necessary for CRM1 export activity [21]. However, while the allosteric regulation of NES binding and release by CRM1 is well established [11,12], there is no structural evidence of conformational changes in CRM1 elicited by S1055 phosphorylation. Furthermore, as previously pointed out [49], XPO1 is an essential gene while STK38 is not, which argues against the proposed notion that STK38 is a necessary activator of CRM1 function. Our results here do not rule out the possibility that S1055 phosphorylation modulates certain aspects of CRM1 activity, but clearly indicate that this modification is not generally required for CRM1 function. In fact, we show that the non-phosphorylatable mutant YFP-CRM1*S1055A retains the ability to properly localize to CB and nucleolus, and is fully able to re-export RanBP1 and p65 to the cytoplasm in LMB-treated cells.

Our final goal was to independently validate previous findings with a mutation that replaces the Q742 residue in human CRM1 with its counterpart in yeast CRM1 (T753). The Q742T mutation has been reported to abrogate the ability of human CRM1 to re-export of RanBP1 and p65 to the cytoplasm in HeLa cells depleted of endogenous CRM1 by siRNA-mediated silencing [17]. Applying a similar rescue strategy (but using LMB to block endogenous CRM1 instead of silencing to reduce its levels) we obtained strikingly different results: YFP-CRM1*Q742T behaved essentially like wild type YFP-CRM1* in terms of intranuclear localization and cargo re-export activity. Thus, our findings do not support previous claims that Q742 represents a function-defining residue for human CRM1. The reason for these discrepant results is unclear and further experiments should clarify this controversy. However, it must be noted that we ensured equal expression levels of the different mutants when comparing their export function, an important control that was not described in the previous report [17].

## 5. Conclusions

Overall, our effort to further characterize a set of mutations targeting key CRM1 residues provides new insights into an understudied aspect of CRM1 biology, its intranuclear localization, and highlights several important issues related to CRM1 function and regulation that need to be addressed in more detail.

## Data Availability

The original contributions presented in this study are included in the article/Supplementary Materials. Further inquiries can be directed to the corresponding author.

## Supplementary Materials

The following supporting information can be downloaded at: https://www.mdpi.com/article/10.3390/biom14121578/s1, Figure S1: Endogenous CRM1 does not accumulate in the Cajal body of untreated HEK293T cells, but relocates to the nucleolus upon ActD treatment. Figure S2: Evaluating the localization to the nucleolus of the different YFP-CRM1 variants using p14^ARF^ as a nucleolar marker. Figure S3: Intranuclear localization of a subset of non-LMB resistant CRM1 variants in untreated HeLa cells. Figure S4: CRM1 mutations differently alter the relocation of the receptor to the nucleolus in ActD-treated HEK293T cells. Figure S5: YFP-CRM1* expression reverts the nuclear accumulation of p65 in LMB-treated, but not in TNFα-treated HeLa cells. Table S1: Results of individual experiments carried out to derive the Cajal body localization score (CBscore) and nucleolar relocation score (NOLscore) of the different CRM1 variants. Table S2: Summary of the results of the independent experiments carried out to determine the ability of the different CRM1 variants to mediate nuclear export of endogenous RanBP1 and p65 in HeLa and HEK293T cells.
