# Supplementary material for: Mutations of Key Functional Residues in CRM1/XPO1 Differently Alter Its Intranuclear Localization and the Nuclear Export of Endogenous Cargos
Source: Biomolecules. 2024 Dec 10;14(12):1578. doi: 10.3390/biom14121578 (PMC11674046; doi:10.3390/biom14121578)
Supplement: Supplementary file 1 [file biomolecules-14-01578-s001.zip › Omaetxebarria et al. Supplementary Figure S1.pdf]

Supplementary Figure S1

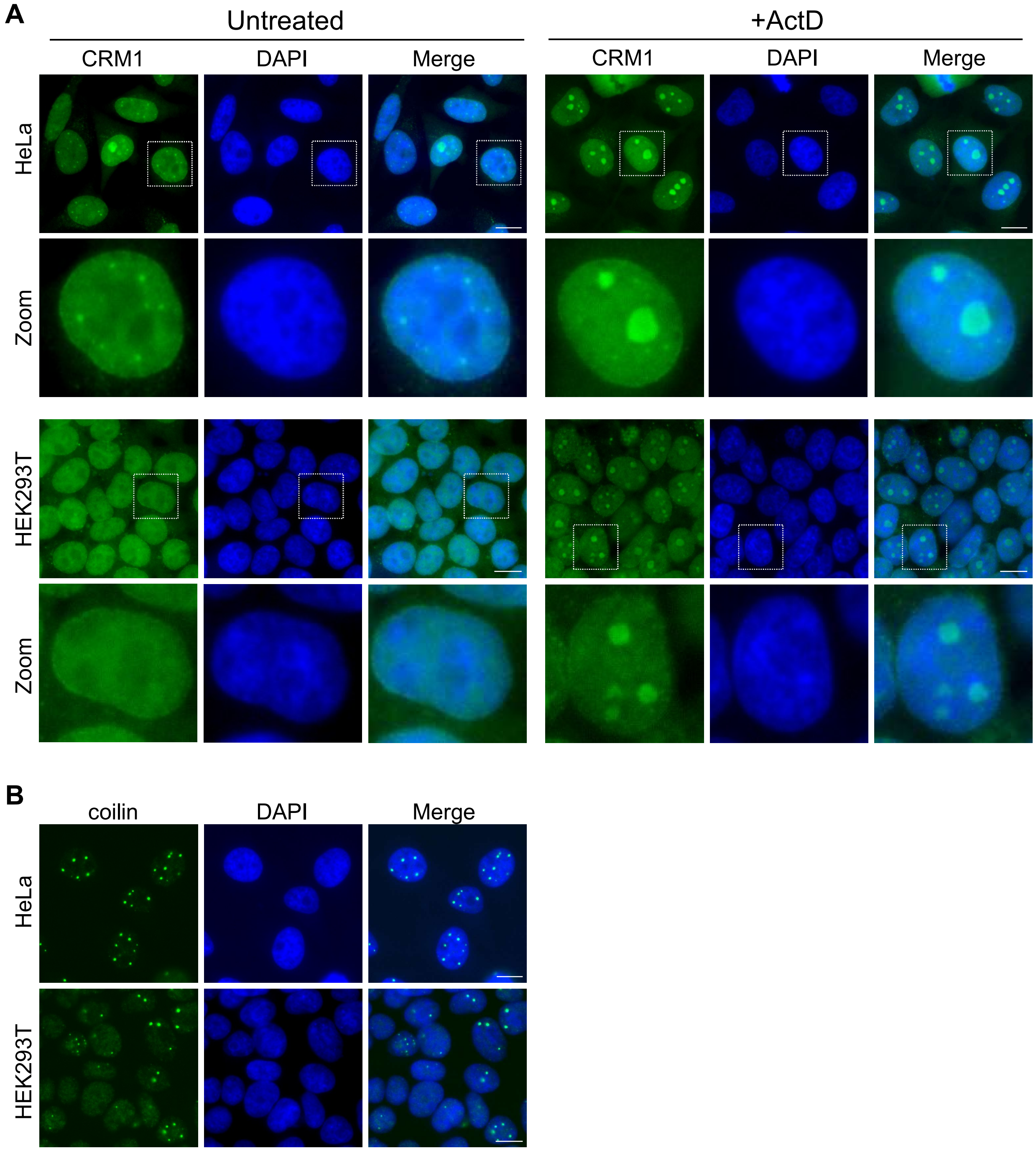

Supplementary Figure S1. Endogenous CRM1 does not accumulate in the Cajal body of untreated HEK293T cells, but relocates to the nucleolus upon ActD treatment.

A. Fluorescence microscopy images showing representative examples of the localization of endogenous CRM1 in untreated or ActD-treated HeLa cells (upper set of panels) and HEK293T cells (lower set of panels). Zoom panels show magnification of one selected nucleus (white square). B. Fluorescence microscopy images showing representative examples of the localization of the Cajal body marker coilin in HeLa and HEK293T cells. DAPI was used to stain the nucleus. Scale bars represent 10 µm.
