# Supplementary material for: Mutations of Key Functional Residues in CRM1/XPO1 Differently Alter Its Intranuclear Localization and the Nuclear Export of Endogenous Cargos
Source: Biomolecules. 2024 Dec 10;14(12):1578. doi: 10.3390/biom14121578 (PMC11674046; doi:10.3390/biom14121578)
Supplement: Supplementary file 1 [file biomolecules-14-01578-s001.zip › Omaetxebarria et al. Supplementary Figure S2.pdf]

Supplementary Figure S2

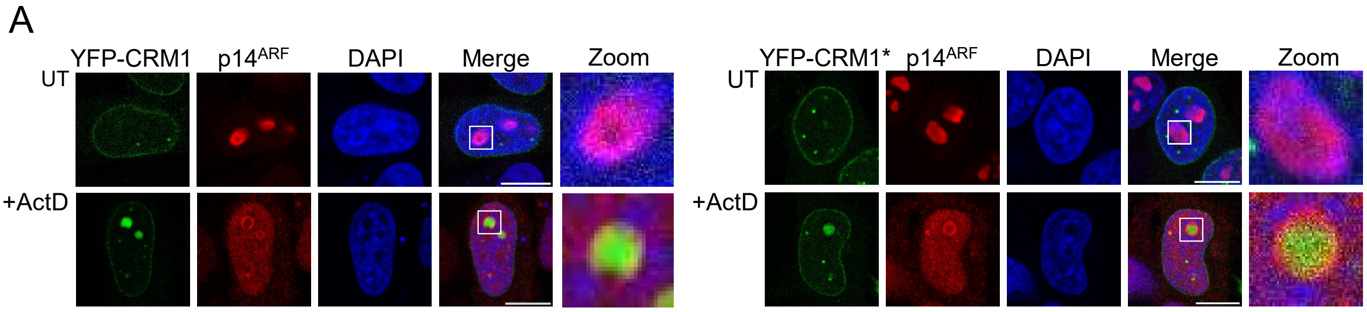

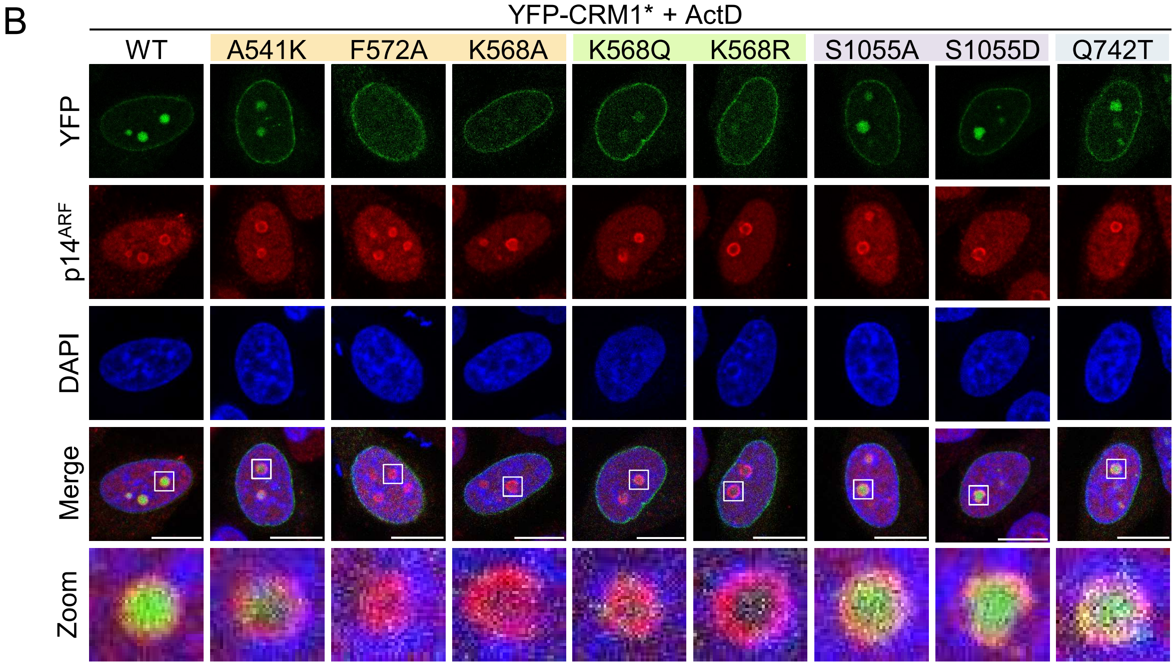

Supplementary Figure S2. Evaluating the localization to the nucleolus of the different YFP-CRM1 variants using p14^{ARF} as a nucleolar marker.

A. Confocal microscopy images showing that YFP-CRM1 and YFP-CRM1* relocalize to the nucleolus of HeLa cells upon ActD treatment (100 ng/mL for 3h). In these experiments, endogenous p14^{ARF} was used as a nucleolar marker. This protein strongly localizes to the nucleus in untreated cells (UT), and largely redistributes to nucleoplasm in cells treated with ActD. B. Confocal microscopy images showing representative examples of the co-localization of the different YFP-CRM1* mutants with endogenous p14^{ARF}. Zoom images show magnification of one selected nucleolus (white square). DAPI was used to stain the nuclei, and the scale bar represents 10 µm.
