# Supplementary material for: Mutations of Key Functional Residues in CRM1/XPO1 Differently Alter Its Intranuclear Localization and the Nuclear Export of Endogenous Cargos
Source: Biomolecules. 2024 Dec 10;14(12):1578. doi: 10.3390/biom14121578 (PMC11674046; doi:10.3390/biom14121578)
Supplement: Supplementary file 1 [file biomolecules-14-01578-s001.zip › Omaetxebarria et al. Supplementary Figure S3.pdf]

Supplementary Figure S3

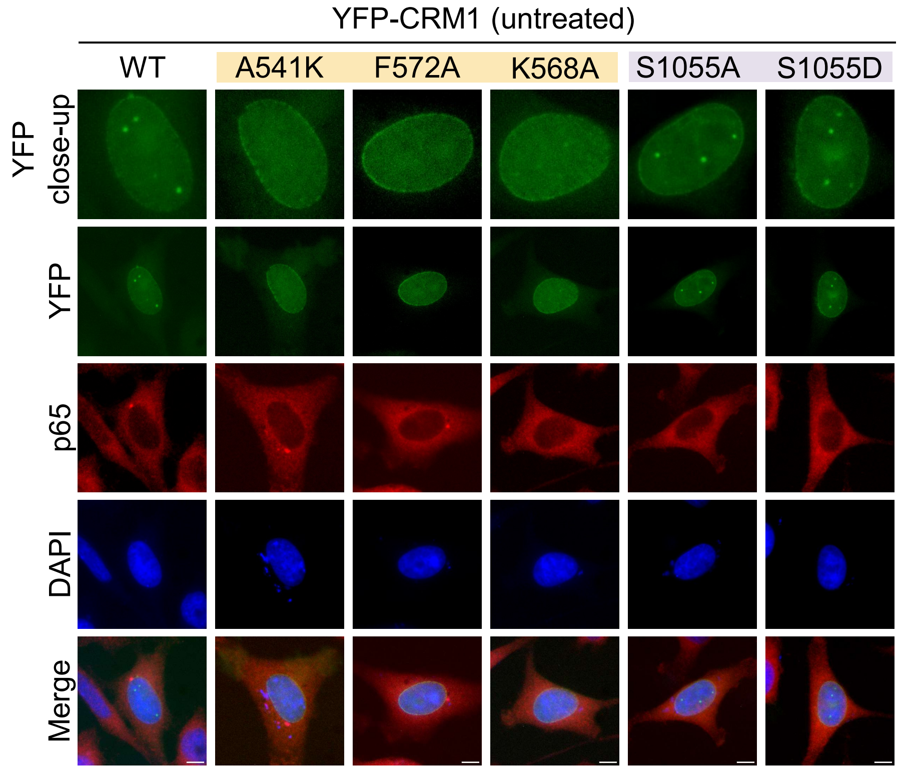

Supplementary Figure S3. Intranuclear localization of a subset of non-LMB resistant CRM1 variants in untreated HeLa cells.

Fluorescence microscopy images showing representative examples of the intranuclear localization of non-LMB resistant YFP-CRM1 variants (not bearing the C528S mutation) and the localization of endogenous p65 in these cells. Consistent with our findings with the C528S-bearing mutant counterparts, the localization of CB is evident in the case of the wild type YFP-CRM1 protein and the phosphorylation site mutants (S1055A and S1055D), but is largely abrogated in NES binding groove mutants (A541K, F572A and K568A). Endogenous p65 is localized to the cytoplasm in untreated HeLa cells expressing these CRM1 mutants. DAPI was used to stain the nuclei, and the scale bar represents 5μm.
