# Supplementary material for: Mutations of Key Functional Residues in CRM1/XPO1 Differently Alter Its Intranuclear Localization and the Nuclear Export of Endogenous Cargos
Source: Biomolecules. 2024 Dec 10;14(12):1578. doi: 10.3390/biom14121578 (PMC11674046; doi:10.3390/biom14121578)
Supplement: Supplementary file 1 [file biomolecules-14-01578-s001.zip › Omaetxebarria et al. Supplementary Figure S4.pdf]

Supplementary Figure S4

A

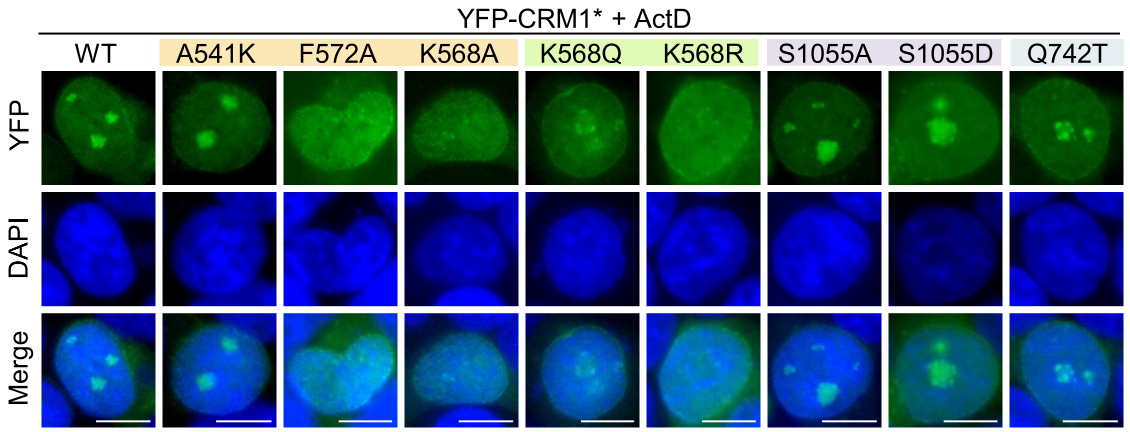

B

YFP-CRM1* variant	Normalized NOLscore
WT	100
A541K	83.88
F572A	10.05
K568A	3
K568Q	26.48
K568R	3.84
S1055A	92.83
S1055D	101.68
Q742T	94.20

Supplementary Figure S4. CRM1 mutations differently alter the relocation of the receptor to the nucleolus in ActD-treated HEK293T cells.
A. Fluorescence microscopy images showing representative examples of the localization of the different YFP-CRM1* mutants in the nucleus of HEK293T cells treated with ActD (100 ng/mL for 3h). Scale bars represent 10 µm. B. Table summarizing the normalized Nucleolar relocation score (Normalized NOLscore) for each variant. This score was derived from the data of a single experiment (analyzing at least 25 cells per condition), by multiplying the percentage of cells with nucleolar localization by the mean nucleolar/nucleoplasm fluorescence intensity ratio. The score of each mutant was normalized to the score of wild type YFP-CRM1*, set at 100.
