# Supplementary material for: Mutations of Key Functional Residues in CRM1/XPO1 Differently Alter Its Intranuclear Localization and the Nuclear Export of Endogenous Cargos
Source: Biomolecules. 2024 Dec 10;14(12):1578. doi: 10.3390/biom14121578 (PMC11674046; doi:10.3390/biom14121578)
Supplement: Supplementary file 1 [file biomolecules-14-01578-s001.zip › Omaetxebarria et al. Supplementary Figure S5.pdf]

Supplementary Figure S5

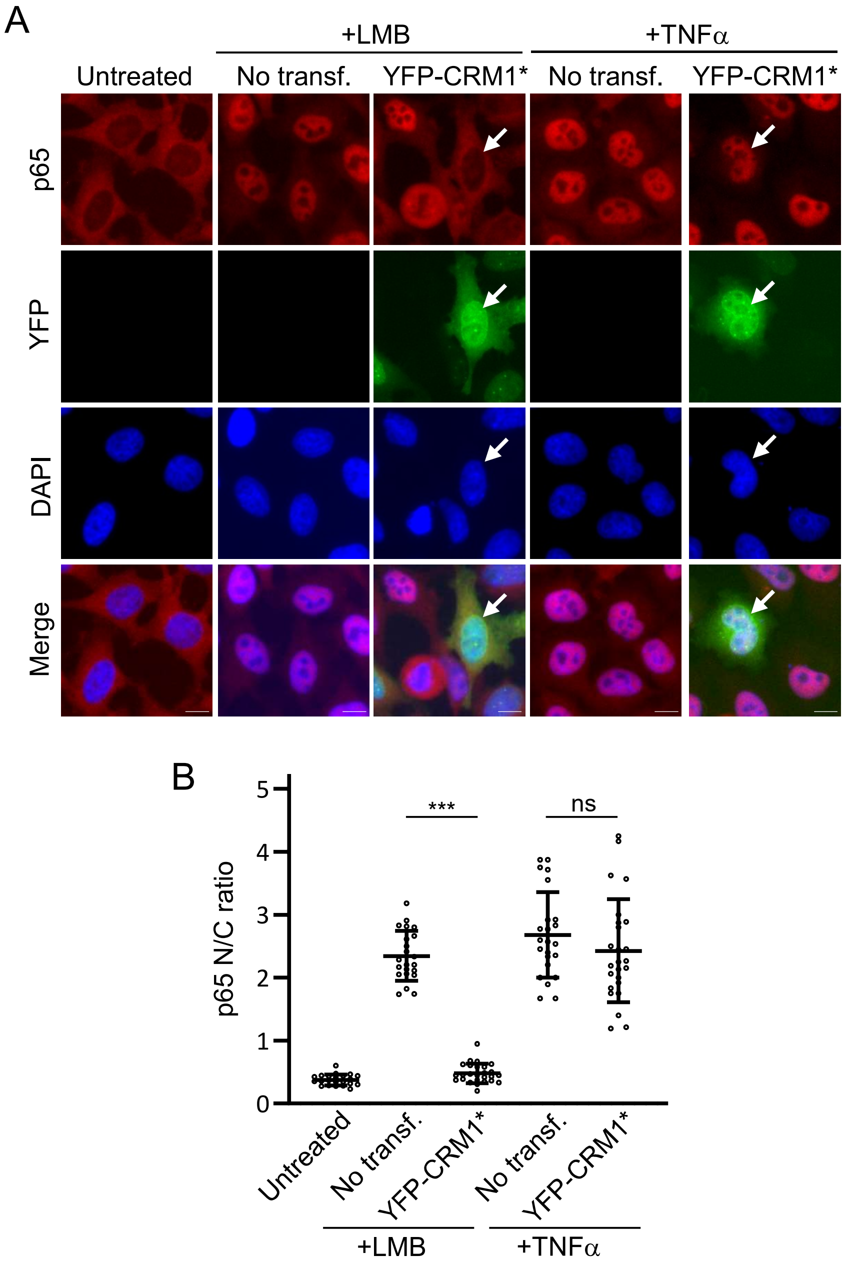

Supplementary Figure S5. YFP-CRM1* expression reverts the nuclear accumulation of p65 in LMB-treated, but not in TNFα-treated HeLa cells.

A. Fluorescence microscopy images showing representative examples of the localization of endogenous p65 in HeLa cells untreated or treated with either LMB (6 ng/mL for 3 h) or TNFα (10 ng/mL for 30 minutes). DAPI was used to visualize the nuclei. Scale bars represent 10 μm. Both drugs induced accumulation of p65 in the nucleus of untransfected cells (No transf.) to a similar extent. Ectopic expression of YFP-CRM1* reverts LMB-induced, but not TNFα-induced nuclear relocation of p65. B. Graph showing nuclear to cytoplasmic (N/C) ratio of the p65 fluorescent signal quantified by image analysis using Fiji. At least 20 cells per condition were scored, each dot representing an individual cell. Mean (horizontal lines) and SD (error bars) are also shown. Student's t test was used to statistically assess the differences between the indicated samples. n.s.: non-significant; (***) $p < 0.001$.
