# Supplementary material for: Mutations of Key Functional Residues in CRM1/XPO1 Differently Alter Its Intranuclear Localization and the Nuclear Export of Endogenous Cargos
Source: Biomolecules. 2024 Dec 10;14(12):1578. doi: 10.3390/biom14121578 (PMC11674046; doi:10.3390/biom14121578)
Supplement: Supplementary file 1 [file biomolecules-14-01578-s001.zip › Omaetxebarria et al. Supplementary Table S1.pdf]

Supplementary Table S1. Results of individual experiments carried out to derive the Cajal body localization score (CBscore) and Nucleolar relocation score (NOLscore) of the different CRM1 variants.

CAJAL BODY LOCALIZATION SCORE (Mean % x Mean ratio of 5 independent experiments)

% = Percentage of cells with Cajal body localization of YFP-CRM1*

Ratio = Cajal body/nucleoplasm fluorescence intensity ratio of YFP-CRM1*

	Experiment #1		Experiment #2		Experiment #3		Experiment #4		Experiment #5		Mean of 5 experiments			
YFP-CRM1*	%	Ratio	%	Ratio	%	Ratio	%	Ratio	%	Ratio	%	Ratio	CBscore	**Normalized CBscore**
WT	98.1	3.8	96.2	3.25	95.2	3.07	96	2.94	91.3	3.07	95.36	3.23	307.63	**100**
A541K	11.6	1.7	3.2	1.7	0	n.a.	12.8	1.55	2.7	2	6.06	1.74	10.53	**3.42**
F572A	52.4	2.04	16.7	1.6	2.67	1.7	13.6	1.49	10.77	1.63	19.23	1.69	32.53	**10.57**
K568A	31.2	1.62	79.4	1.6	41.4	1.5	37.2	1.46	41.77	1.67	46.25	1.56	71.97	**23.39**
K568Q	39.4	1.65	22.5	1.55	18.1	1.23	20	1.67	15.2	2.05	23.04	1.63	37.55	**12.20**
K568R	40	1.78	31.8	1.32	16.7	1.25	28.8	1.44	18.2	1.76	27.1	1.51	40.92	**13.30**
S1055A	97.7	3.03	94.8	3.05	95	2.39	100	3.08	92.54	2.44	96.01	2.80	268.63	**87.32**
S1055D	96.6	3.2	98.1	3.13	85.7	2.38	98	2.55	91.25	3.19	93.93	2.89	271.46	**88.24**
Q742T	97.4	4.3	95.1	4	84.2	3.15	97	2.9	88.8	3.04	92.5	3.48	321.72	**104.58**

NUCLEOLAR RELOCATION SCORE (Mean % x Mean ratio of 4 independent experiments)

% = Percentage of cells with nucleolar localization of YFP-CRM1*

Ratio = Nucleolus/nucleoplasm fluorescence intensity ratio of YFP-CRM1*

	Experiment #1		Experiment #2		Experiment #3		Experiment #4		Mean of 4 experiments			
YFP-CRM1*	%	Ratio	%	Ratio	%	Ratio	%	Ratio	%	Ratio	NOLscore	**Normalized NOLscore**
WT	100	3.1	95.5	2.4	91	2.87	98.3	2.6	96.2	2.74	263.82	**100**
A541K	87.5	2.7	92.6	2.1	94.3	2.12	95.1	2.4	92.37	2.33	215.23	**81.58**
F572A	12.5	1.48	0	n.a.	0	n.a.	4.2	2.8	4.17	2.14	8.93	**3.38**
K568A	33.3	1.44	0	a.a.	0	n.a.	0	n.a.	8.32	1.44	11.99	**4.54**
K568Q	68.7	1.54	13.2	1.24	42.6	1.35	17.3	1.35	35.45	1.37	48.56	**18.40**
K568R	44.4	1.51	6.25	1.4	9	1.25	0	n.a.	14.91	1.39	20.68	**7.83**
S1055A	96	2.9	95.7	2.51	100	2.39	92.1	2.31	95.95	2.53	242.51	**91.92**
S1055D	95.2	3.2	100	2.42	100	2.44	89	2	96.05	2.51	241.56	**91.56**
Q742T	100	3.1	100	2.41	95.8	2.56	89.8	2.56	96.4	2.66	256.18	**97.10**
