# Supplementary material for: Mutations of Key Functional Residues in CRM1/XPO1 Differently Alter Its Intranuclear Localization and the Nuclear Export of Endogenous Cargos
Source: Biomolecules. 2024 Dec 10;14(12):1578. doi: 10.3390/biom14121578 (PMC11674046; doi:10.3390/biom14121578)
Supplement: Supplementary file 1 [file biomolecules-14-01578-s001.zip › Omaetxebarria et al. Supplementary Table S2.pdf]

Supplementary Table S2. Summary of the results of the independent experiments carried out to determine the ability of the different CRM1 variants to mediate nuclear export of endogenous RanBP1 and p65 in HeLa and HEK293T cells. For each experiment, the mean expression level of each CRM1 variant and the mean nuclear to cytoplasmic (N/C) or nuclear to total (N/T) ratio of the marker is indicated. n.t.= not tested (CRM1 variant not included in the experiment).

RanBP1 N/C ratio in Hela cells

	Experiment #1		Experiment #2		Experiment #3		Experiment #4		Experiment #5	
YFP-CRM1*	CRM1 expression	RanBP1 N/C ratio	CRM1 expression	RanBP1 N/C ratio	CRM1 expression	RanBP1 N/C ratio	CRM1 expression	RanBP1 N/C ratio	CRM1 expression	RanBP1 N/C ratio
WT	22.45	0.91	33.70	1.20	27	1.44	34.7	1.50		
A541K	n.t.	n.t.	33.33	2.04	27.1	2.62	34	3.45		
F572A	22.25	3.33	33.11	1.99	27.1	2.64	34.64	3.18		
K568A	n.t.	n.t.	33.02	1.96	27.3	2.50	34.55	3.53		
K568Q	n.t.	n.t.	33.54	1.93	27.2	2.30	34.56	2.96		
K568R	n.t.	n.t.	33.15	1.82	27.1	2.10	34	3.49		
S1055A	22.66	1.11	33.68	1.24	27.3	1.50	34.52	1.2		
S1055D	22.39	1.17	33.12	1.37	27.3	1.34	34.10	1.36		
Q742T	n.t.	n.t.	33.30	1.36	n.t.	n.t.	34.05	1.51		

RanBP1 N/T ratio in HEK293T cells

	Experiment #1		Experiment #2		Experiment #3		Experiment #4		Experiment #5	
YFP-CRM1*	CRM1 expression	RanBP1 N/T ratio	CRM1 expression	RanBP1 N/T ratio	CRM1 expression	RanBP1 N/T ratio	CRM1 expression	RanBP1 N/T ratio	CRM1 expression	RanBP1 N/T ratio
WT	46.3	0.93	46.4	0.80	50.78	0.73				
A541K	46.2	1.83	46.3	1.60	50	1.41				
F572A	46.4	1.43	46.1	1.57	49.85	1.45				
K568A	46.2	1.62	46.4	1.46	50.09	1.35				
K568Q	46.3	1.17	46.4	1.43	50.82	1.25				
K568R	46.2	1.43	46.2	1.51	49.52	1.29				
S1055A	46.2	0.95	46.4	1.05	49.71	0.76				
S1055D	46.1	0.83	46.4	1.02	50.66	0.74				
Q742T	46.4	0.97	46.2	1.01	49.45	0.75				

p65 N/C ratio in Hela cells

	Experiment #1		Experiment #2		Experiment #3		Experiment #4		Experiment #5	
YFP-CRM1*	CRM1 expression	p65 N/C ratio	CRM1 expression	p65 N/C ratio	CRM1 expression	p65 N/C ratio	CRM1 expression	p65 N/C ratio	CRM1 expression	p65 N/C ratio
WT	33.42	1.19	21.1	0.49	22.86	0.59	31.55	0.97	33.33	1.31
A541K	33.36	2.46	22.4	2.87	22.41	2.1	32.04	3.85	n.t.	n.t.
F572A	33.33	0.71	21	0.33	22.73	0.52	n.t.	n.t.	n.t.	n.t.
K568A	33.63	2.33	n.t.	n.t.	n.t.	n.t.	32.1	3.30	33.23	2.83
K568Q	33.55	2.18	n.t.	n.t.	n.t.	n.t.	33.1	3.34	33.04	3.25
K568R	33.61	1.87	n.t.	n.t.	n.t.	n.t.	32.13	2.75	33.4	2.85
S1055A	33.61	0.93	16.3	0.6	n.t.	n.t.	n.t.	n.t.	33.23	1.36
S1055D	33.59	0.93	21.56	0.49	n.t.	n.t.	n.t.	n.t.	33.83	1.38
Q742T	33.63	0.95	n.t.	n.t.	n.t.	n.t.	n.t.	n.t.	33.62	1.33

p65 N/T ratio in HEK293T cells

	Experiment #1		Experiment #2		Experiment #3		Experiment #4		Experiment #5	
YFP-CRM1*	CRM1 expression	p65 N/T ratio	CRM1 expression	p65 N/T ratio	CRM1 expression	p65 N/T ratio	CRM1 expression	p65 N/T ratio	CRM1 expression	p65 N/T ratio
WT	42.72	0.63	30,56	0,62	25.99	0.54	47	0.58	47.32	0.58
A541K	42.88	1.15	30,68	0,94	27.38	0.99	47,91	1.09	n.t.	n.t.
F572A	42.66	0.39	30,5	0,53	25.33	0.42	47.15	0.51	n.t.	n.t.
K568A	42.75	0.96	n.t.	n.t.	25.73	0.89	47.37	1.15	n.t.	n.t.
K568Q	42.11	1.29	n.t.	n.t.	26.75	1.11	47.85	1.12	n.t.	n.t.
K568R	42.28	1.07	n.t.	n.t.	25.77	1.04	47.52	1.00	n.t.	n.t.
S1055A	42.60	0.58	n.t.	n.t.	n.t.	n.t.	47.09	0.61	46.95	0.63
S1055D	42.28	0.48	n.t.	n.t.	n.t.	n.t.	47.5	0.57	47.4	0.57
Q742T	42.26	0.66	n.t.	n.t.	26.12	0.61	47.08	0.59	47.52	0.60
